# A novel flexible microfluidic meshwork to reduce fibrosis in glaucoma surgery

**DOI:** 10.1371/journal.pone.0172556

**Published:** 2017-03-16

**Authors:** Behzad Amoozgar, Xiaoling Wei, Jun Hui Lee, Michele Bloomer, Zhengtuo Zhao, Paul Coh, Fei He, Lan Luan, Chong Xie, Ying Han

**Affiliations:** 1 Department of Ophthalmology, University of California, San Francisco, California, United States of America; 2 Department of Biomedical Engineering, the University of Texas at Austin, Austin, Texas, United States of America; 3 Department of Physics, the University of Texas at Austin, Austin, Texas, United States of America; Harvard Medical School, UNITED STATES

## Abstract

**Purpose/Relevance:**

Fibrosis and hence capsule formation around the glaucoma implants are the main reasons for glaucoma implant failure. To address these issues, we designed a microfluidic meshwork and tested its biocompatibility in a rabbit eye model. The amount of fibrosis elicited by the microfluidic meshwork was compared to the amount elicited by the plate of conventional glaucoma drainage device.

**Methods:**

Six eyes from 3 New Zealand albino rabbits were randomized to receive either the novel microfluidic meshwork or a plate of Ahmed glaucoma valve model PF7 (AGV PF7). The flexible microfluidic implant was made from negative photoresist SU-8 by using micro-fabrication techniques. The overall size of the meshwork was 7 mm × 7 mm with a grid period of 100 μm. Both implants were placed in the subtenon space at the supratemporal quadrant in a standard fashion. There was no communication between the implants and the anterior chamber via a tube. All animal eyes were examined for signs of infection and implant erosion on days 1, 3, 7, and 14 and then monthly. Exenterations were performed in which the entire orbital contents were removed at 3 months. Histology slides of the implant and the surrounding tissues were prepared and stained with hematoxylin-eosin. Thickness of the fibrous capsules beneath the implants were measured and compared with paired student’s t-test between the two groups.

**Results:**

The gross histological sections showed that nearly no capsule formed around the microfluidic meshwork in contrast to the thick capsule formed around the plate of AGV PF7. Thickness of the fibrotic capsules beneath the AGV PF7 plate from the 3 rabbit eyes was 90μm, 82μm, and 95 μm, respectively. The thickness at the bottom of fibrotic capsules around the new microfluidic implant were 1μm, 2μm, and 1μm, respectively. The difference in thickness of capsule between the two groups was significant (*P* ***=*** 0.002). No complications were noticed in the 6 eyes, and both implants were tolerated well by all rabbits.

**Conclusion:**

The microfluidic meshwork elicited minimal fibrosis and capsule formation after 3-months implantation in a rabbit model. This provides promising evidence to aid in future development of a new glaucoma drainage implant that will elicit minimal scar formation and provide better long-term surgical outcomes.

## Introduction

Glaucoma is the leading cause of irreversible blindness in the world.[1] To date, controlling intraocular pressure (IOP) remains the primary treatment option. [2] Glaucoma surgery is commonly considered when glaucoma eye drops and laser therapies fail to lower IOP. [3]The fundamental concept of glaucoma surgery is to artificially create an additional pathway for aqueous humor (AH) outflow, therefore lowering IOP.

Trabeculectomy, glaucoma drainage implants (GDIs) and minimally invasive glaucoma surgeries (MIGS) are the currently available surgical treatment for glaucoma. Trabeculectomy is the standard surgical approach to treat adult primary glaucoma, but its success in children and certain glaucoma populations is limited.[4–7] GDIs were developed to treat patients with secondary glaucoma, pediatric glaucoma, and refractory glaucoma after failed trabeculectomy. Unfortunately, the long-term outcome of GDIs has not been satisfactory, largely due to fibrotic encapsulation of the implant that impedes the drainage of fluid.[8,9] MIGS is a relatively new approach with a superior safety profile, but mainly targets mild to moderate glaucoma with the goal of reducing the use of glaucoma drops. Failure, for all three types of glaucoma surgeries, results from the natural healing process of the human body that attempts to repair and close the new openings either at episcleral tissue for trabeculectomy [10,11] or around the implants for GDIs [12–14] or MIGS.[15]

**Fig 1:**
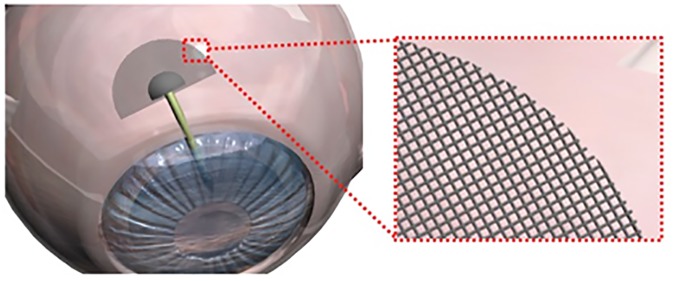
Proposed concept of a new GDI. Plate of the GDI is replaced by the microfluidic meshwork.

Thus, there is a critical need to develop an implant that can sustain the aqueous outflow while preventing obstruction due to fibrosis–the key to long-term functionality. Here, we present the concept for a modified GDI (**Fig 1**). The device design is based on that of the conventional GDI, however, we replaced the solid plate with a microfluidic meshwork in the fluid drainage region. The design of the meshwork was inspired by recently developed brain implants that can suppress chronic foreign body reactions.[16] We have incorporated two key features of the brain implants into the microfluidic meshwork design. Firstly, it consists of interconnected, cellular-dimensioned microfluidic channels that can conduct fluid. Secondly, it is ultra-flexible and conforms to the curvature and movement of the eye tissue after implantation. We hypothesize that these two features combined minimize fibrotic tissue formation around the meshwork, and therefore reduce the risk of failure of the drainage implants. In this work, as the initial test of the viability of this concept, we investigated the chronic tissue reactions to the implanted microfluidic meshwork alone where no tube was connected to the meshwork in the rabbit model. We used the plate of the conventional GDI, Ahmed glaucoma valve (AGV, PF7 model), as the control.

## Material and methods

### Meshwork fabrication

**Fig 2:**
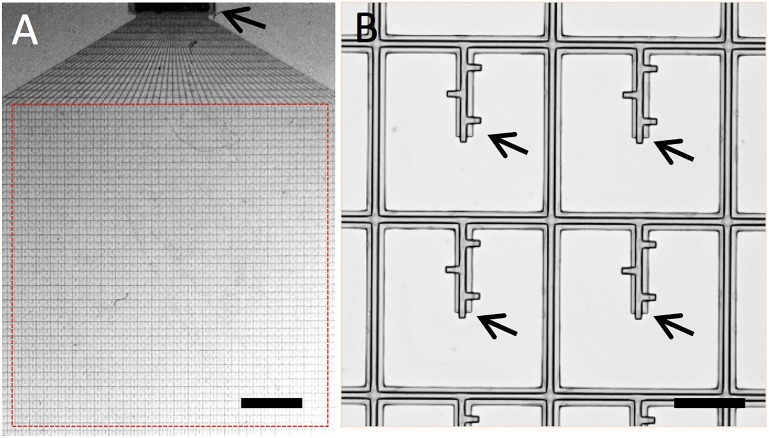
Images of the microfluidic meshwork. A. on a substrate. B. Zoom-in view of the mesh grids. Arrows denote the fluid outlets. Scale bars: 500 μm and 100 μm.

The drainage devices were fabricated using photolithography techniques similar to those that were demonstrated previously.[17] The fabrication was done on silicon wafers with a nickel-releasing layer. Briefly, microchannel walls were patterned with negative photoresist SU-8 and the microchannels were formed by sacrificial photoresist (LOR 5A and AZ1505, Microchem, Westborough, MA). The meshwork had an overall area of 7mm × 7mm and a grid period of 100μm. The thickness of the meshwork was 4 μm (**Fig 2**). The microfluidic channels had outer diameters of 20 μm and inner diametersof 8μm. These parameters were determined according to finite element simulations to provide sufficient AH outflow (2 μL/min at 10 mmHg). After being released from the substrate, the meshworks were washed and stored in buffer solution prior to autoclave and implantation. The design, fabrication and simulation of the meshwork is documented elsewhere in details.[18]

### Animal preparation and standard surgical implantation

**Fig 3:**
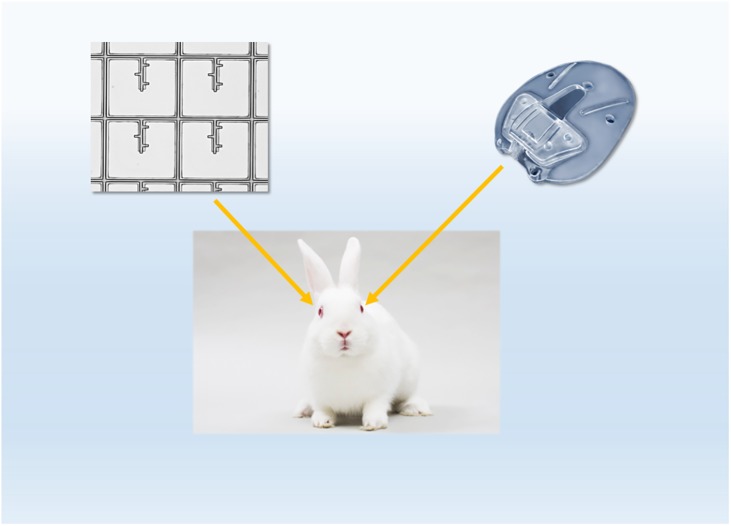
Implantation in rabbit eyes. One eye was assigned to the conventional AGV PF7 silicone plate and the other to the microfluidic meshwork. No tube was connected with either AGV PF7 plate or the microfluidic meshwork.

Three healthy Albino New Zealand rabbits (12–14 weeks old, and weighing 2–3 kg) were purchased and maintained at the Laboratory Animal Resource Center (LARC) at the University of California, San Francisco (UCSF). The Institutional Animal Care and Use Committee (IACUC) of UCSF approved the study. All animals were treated in accordance with the ARVO Statement for the Use of Animals in Ophthalmic and Vision Research. For each rabbit, the eyes were randomized to have one assigned to the current AGV PF7 silicone plate (no tube connected) and the other to the microfluidic meshwork (**Fig 3**). The surgery was performed in standard fashion in the animal microsurgery suite at UCSF. Under an operating microscope, the rabbits were anesthetized using an intramuscular injection of a mixture of ketamine hydrochloride (50 mg/kg) and xylazine hydrochloride (10 mg/kg), followed by mask anesthesia of isoflurane (2–4%). All efforts were made to minimize suffering of the animals. Both eyes were then prepared with povidone-iodine. For each eye, 6–0 Vicryl suture was passed through the supratemporal limbus to rotate the eye downward. Conjunctival peritomy was performed at the limbus in the supratemporal quadrant, followed by posterior dissection in the same plane. The flexible microfluidic meshwork was placed without suture and the AGV FP7 plate was sutured with 9–0 nylon sutures onto the episcleral surface approximately 6 mm from the limbus. The conjunctiva was closed with interrupted 8–0 vicryl sutures. There was no communication with the anterior chamber with either implant. To facilitate visualization of the microfluidic meshwork, a limbal 10–0 nylon suture was placed at the middle of the microfluidic meshwork. At the end of the surgery, subconjunctival cefazolin 0.1ml was given for antimicrobial prophylaxis. As routine postoperative care, the rabbit eyes were treated with Polymyxin antibiotic drops for one week and prednisolone acetate 1% drops starting with 3 times a day and then tapered every 3 days. All animal eyes were examined for signs of infection and plate erosion on days 1, 3, 7, and 14 and monthly thereafter for up to 3 months. Three months after surgery, the rabbits were euthanized by intravenous injection of potassium chloride or sodium pentobarbital after being anesthetized by isoflurane or ketamine/xylazine combination.

### Histology preparation

Three months after surgery, the rabbits were sacrificed and exenterations were performed in which the entire orbital contents were removed. Precautions were taken not to disturb the implants. After being fixed with 10% formalin, the eyes were dissected. Histology slides of the implant and the surrounding tissues were prepared and stained with hematoxylin-eosin (HE). The histological sections were examined and measured using light microscopy by a pathologist who was blinded to the different groups. Capsule thickness at the bottom of the plate was measured for each eye.

### Statistical considerations

Paired student’s t-test was applied to compare the difference in the thickness of fibrous capsule between the microfluidic meshwork and conventional AGV PF7 plate groups. The rate of infection/plate erosion and any other notable side effects were compared between the two groups using the Fisher exact test.

## Results

Six eyes from 3 New Zealand rabbits underwent implantation of the plate of AGV PF7 in one eye and the microfluidic meshwork in the other in a randomized fashion. Two left eyes and one right eye received AGV PF7 while the other two right eyes and one left eye received the microfluidic meshwork. There was no tube connected to either AGV PF7 or the microfluidic meshwork. No significant complications were noticed during the implantation of both AGV PF7 and the microfluidic meshwork.

**Fig 4:**
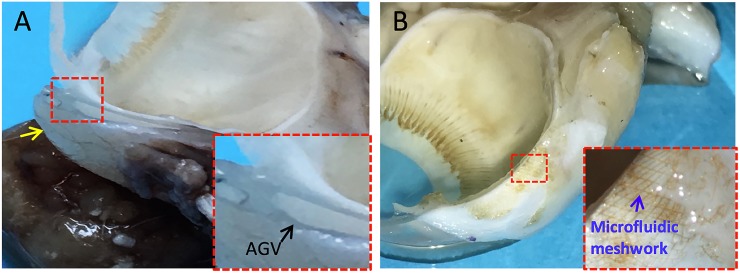
Gross section of tissue reactions to the microfluidic meshwork in comparison with AGV 3 months post implantation. **A.** AGV **B.** microfluidic meshwork. Inset figures are magnified views of the microfluidic meshwork.

**Fig 5:**
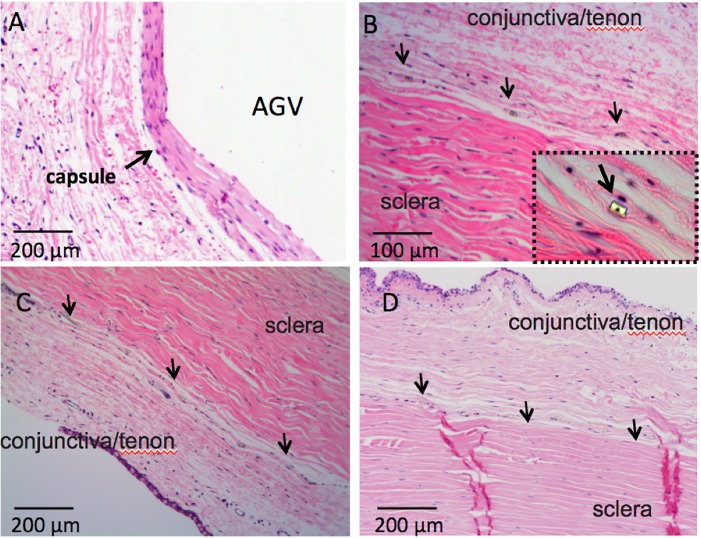
Histological study of tissue reaction to the microfluidic meshwork in comparison with AGV 3 months post implantation. **A.** capsule beneath the plate of AGV; **B.** minimal reaction to the meshwork in rabbit 1, inset figure is a magnified view to a single channel of the meshwork (400x); **C.** minimal reaction to the meshwork in rabbit 2; **D.** minimal reaction to the meshwork in rabbit 3. Arrows in B, C and D is to delineate the meshwork.

During the postoperative visits, there were no signs of infections, inflammation or erosion in any eye. All rabbits tolerated both types of implants well. After 3 months, exenterations were performed and the entire orbits were processed for HE staining. As shown in **Fig 4**, we observed that a thick capsule had formed around the plate of the AGV PF7 while nearly no capsule formed around the microfluidic meshwork (brown). Average thickness of the fibrotic capsules beneath the AGV PF7 and the microfluidic meshworks were 89 ± 6.6μm and 1.3 ± 0.6μm, respectively. There was a significant difference between the two groups (*P* = 0.002, **Fig 5**).

**Fig 6:**
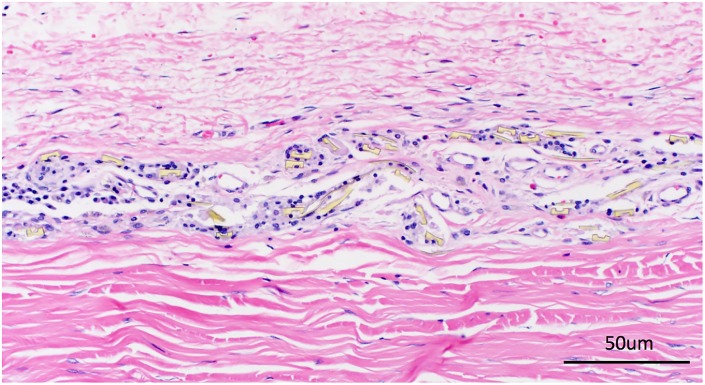
Histological study of tissue reaction to the stacked microfluidic meshwork. In the region where the meshwork has stacked into multiple layers during surgery, increased inflammatory reaction was noticed compared to the single layer region.

During further inspection of all the histological slides of meshwork implants, we noticed that some inflammatory cells accumulated in the region where the meshwork had stacked into multiple layers during surgery (< 10% of the total area, **Fig 6**). This was not observed around monolayer meshwork.

## Discussion

We presented here a modified concept of a GDI, in which the traditional plate of the GDI would be replaced by microfluidic meshwork. In this study, we tested our hypothesis that a properly designed meshwork can significantly suppress fibrotic tissue formation. In clear contrast to conventional implants, the microfluidic meshwork demonstrated excellent biocompatibility, evidenced by nearly no scar tissue and minimal inflammation.

Scar formation is the key obstacle in the surgical management of glaucoma. Modifications to the surgical technique as well as addition of intraoperative and postoperative medications have been studied in an effort to modulate fibrosis and promote long-term success with variable results.[19–27] Mitomycin-C and 5-fluorouracil have been used intraoperatively and postoperatively to reduce inflammation.[19] However, the use of these agents has been associated with significant complications.[20,21] While corticosteroids offer reduced side effects, they are less potent and fail to provide significant improvement in long-term IOP reduction.[26] Modifications to the material comprising glaucoma surgical devices have also been explored. Studies that compared conventional silicone flexible GDI with polypropylene rigid GDI have shown a lower rate of encapsulation and higher success rates with the flexible plate.[28] Several novel designs of glaucoma surgical devices aimed at reducing scar formation have been tested, including a MMC-coated valve, Ferrofluid valve, expanded polytetrafluoroethylene enclosed Ahmed, and Ahmed Glaucoma Valve with Adjunctive Amniotic Membrane.[29–32] These modifications and designs have been shown to decrease the amount of fibrosis and scar tissue when compared to conventional glaucoma devices but still resulted in significant capsule formation.[31,32]

The most active phase of capsule formation occurs in the 3 months following implantation.[8] Although many theories have been proposed to explain the increased rate of encapsulation with glaucoma surgical devices, including those on the physical profile of the implant (e.g. size and material) and fibrosis stimulation through early exposure to inflammatory mediators,[28,33,34] the fundamental mechanisms of these tissue reactions are not clearly understood. Nonetheless, in a series of recent works,[35] neural probes were successfully engineered to suppress tissue reactions by addressing two important problems. First, the mechanical mismatch between the tissue and the implant gives rise to interfacial forces that constantly elicit tissue reactions. Second, the presence of the solid implant interrupts the cellular and vascular networks at the implant site.[9] In order to achieve optimal biocompatibility, these two issues must be addressed through substantial changes to the implant’s mechanical properties and geometric structure. Hence, the implant was designed as a network of ultra-flexible interconnected cellular-sized channels in order to optimize its fluidic conductance while introducing minimum perturbation to the cellular and vascular processes at the implant site.

Additionally, during the histological examination of the dissected specimens, we noticed some inflammatory cell activation at the edge of the meshwork implant wherever the meshwork was folded or stacked into layers during the surgery. These regions were less than 10% of the total meshwork area. We postulated that when the meshwork was folded into multiple layers, its flexibility and hence its biocompatibility with eye tissue was compromised. This led to increased inflammatory cell activation and aggregation. This was not noticed in the majority of the areas where a single layer was maintained.

We chose a New Zealand rabbit model for this study because this model has been previously used to study the effects of various biomaterials as well as newly-designed glaucoma surgical devices on the degree of fibrosis.[36,37] Subsequent clinical studies were concordant and confirmed the applicability of the rabbit model.[23,38] The plate of AGV FP7, one of the most commonly used glaucoma surgical device in practice, was chosen as the control group. In addition to its popularity, AGV FP7 has been well characterized in the rabbit model and a large amount of published histological results can serve as reference. In our study, the average capsule thickness under the AGV PF7 plate implant was 89μm, which is comparable with the capsule thickness reported in the literature.[29–32]

One limitation of this study was that we only studied the tissue reactions to non-fluid draining implants. The mechanical mismatch between tissue and implant may not be the only factor affecting fibrosis. It is known that inflamed AH may also lead to inflammation around the implant and elicit tissue reactions,[39] and this study does not account for this. However, our results show that the AGV PF7 plate had a significant amount of fibrotic capsule even without AH flow. This suggests that the implant itself is one of the major, if not the total, cause of fibrosis. Furthermore, inflamed AH can be treated with extensive anti-inflammatory medication, such as steroid eye drops and/or anti-aqueous suppressants to minimize the effect. In the next step, we plan to construct a complete GDI from the microfluidic meshwork and investigate its fluidic dynamics as well as tissue reactions while accounting for the AH flow.

In summary, this study demonstrated that the flexible microfluidic meshwork elicited minimal scar formation after being implanted into rabbit eyes for 3 months. We believe that these encouraging results warrant further development of a new GDI based on the microfluidic meshwork. The resulting GDI may significantly reduce fibrosis around the device and improve long-term success rates of glaucoma surgery across the spectrum.
